# Reversed Conformation and Complete Transposition of All the Thoracic and Abdominal Viscera, in a Man Twenty-one Years of Age

**Published:** 1857-06

**Authors:** Andrew Foster

**Affiliations:** Terre Coupee, St. Joseph County, Indiana


*Reversed conformation and complete transposition of all the Thoracic and Abdominal Viscera, in a man twenty-one years of age.* By Andrew Foster, M.D., of Terre Coupee, St. Joseph County, Indiana.

Terre Coupee, May 7th, 1857.

Prof. N. S. Davis:

*Dear Sir:* The above caption expresses literally the facts of this very singular case; nevertheless to gratify the inquisitive and convince the sceptical, I will particularize as briefly as possible by referring to Wilson's (or any other) Anatomy. Turn to the appropriate chapter; 1st, of the lungs; 2d, of the stomach, duodeum, jejunum, illium, and colan; 3d, the heart and arteries and veins, 4th, the liver; 5th, the spleen; and 6th, the kidneys. Then read, transposing the terms right and left, or in other words, for the word right, wherever it occurs in the text, read left, and for left read right, and you have an accurate description of the anatomical structure, conformation and position of the organs, as revealed in this dissection, excepting only the kidneys;—here Nature seems to have made an effort to preserve the normal relative positions of these organs, and to have succeeded but too well; that on the left side being a little too high, while that on the right is a great deal too low, being entirely within the pelvis. The supra-renal capsule is on a level with the promontory of the sacrum, the lower end of the kidney reaching to the coxyx, and the body lying close along the right side of the sacrum.

This subject died on the 5th instant, after an illness of a few days, of pulmonary congestion. I was called in during his illness, by Dr. Rouse, the attending physician, and observing the heart to beat at a point between the fifth and sixth ribs of the right side, for which phenomenon I was unable to account satisfactorily to either myself or the friends. After death, I asked for and obtained the privilege of an autopsy, which, assisted by Dr. Rouse, we immediately set about executing, twenty hours after dissolution.

This young man was short, heavy set, strong, active, and muscular, of sanguine temperament, with very short neck and capacious chest. Up to his thirteenth year he had been remarkably strong, active, and healthy; but about that age he *began to have fits,* as they expressed it, but what kind they were I am not able to form an opinion from the imperfect description of the symptoms, as detailed by the friends. Lately they had become less frequent, having had but two in the last two years, the last one being about the commencement of the last sickness.

I have preserved the heart for the inspection of the curious, and any further information in regard to the particulars will be cheerfully imparted to any inquirer.

